# Supplementary material for: ST09, A Novel Curcumin Derivative, Blocks Cell Migration by Inhibiting Matrix Metalloproteases in Breast Cancer Cells and Inhibits Tumor Progression in EAC Mouse Tumor Models
Source: Molecules. 2020 Sep 30;25(19):4499. doi: 10.3390/molecules25194499 (PMC7583863; doi:10.3390/molecules25194499)
Supplement: Supplementary file 1 [file molecules-25-04499-s001.pdf]

**Supplementary Materials:**

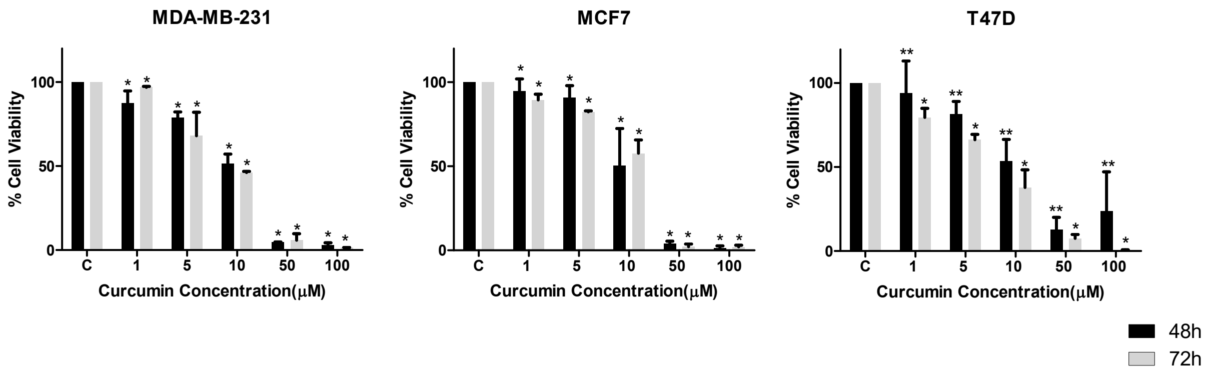


**Supplementary Figure 1: Evaluation of cell viability after Curcumin treatment by LDH Assay**: Bar graph depicting cell viability upon Curcumin treatment on 3 cell lines as tested by LDH assay. All experiments were performed a minimum of three times and the bar graph shows mean ± SEM. Two-way ANOVA test was performed and p value was calculated between control and ST09 treated groups(*p < 0.05, **p < 0.005, ***p < 0.0001,*****p<0.00001).

| Curcumin | |
|---|---|
| 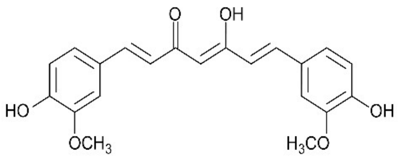 | |
| **Cell lines** | **IC50 (uM)** |
| MDA-MB-231 | 10.53 |
| MCF7 | 13.95 |
| T47D | 10.17 |

**Supplementary Table 1: Structure of Curcumin and IC50 values for the breast cancer cells are tabulated for 48h treatment**
